# The First *Ptilodactyla* Illiger, 1807 (Coleoptera: Dryopoidea: Ptilodactylidae) Described from Eocene Baltic Amber

**DOI:** 10.3390/biology10090877

**Published:** 2021-09-06

**Authors:** Robin Kundrata, Gabriela Packova, Kristaps Kairišs, Andris Bukejs, Johana Hoffmannova, Stephan M. Blank

**Affiliations:** 1 Department of Zoology, Faculty of Science, Palacky University, 17. Listopadu 50, 77146 Olomouc, Czech Republic; gabriela.packova01@upol.cz (G.P.); johana.hoffmannova01@upol.cz (J.H.); 2 Department of Biosystematics, Institute of Life Sciences and Technology, Daugavpils University, Vienības 13, 5401 Daugavpils, Latvia; kr.kairiss@gmail.com (K.K.); carabidae@inbox.lv (A.B.); 3 Senckenberg Deutsches Entomologisches Institut, Eberswalder Strasse 90, 15374 Müncheberg, Germany; Stephan.Blank@senckenberg.de

**Keywords:** beetles, Byrrhoidea, diversity, Elateriformia, fossil, Ptilodactylinae, tertiary, X-ray microcomputed tomography

## Abstract

**Simple Summary:**

Recent advances in computational and tomographic methods have enabled detailed descriptions of fossil specimens embedded in amber. In this study, we used X-ray microcomputed tomography to reconstruct the morphology of a specimen of the beetle family Ptilodactylidae from Eocene Baltic amber. The studied specimen represents a new species of the large and wide-spread genus *Ptilodactyla* Illiger, 1807. It is the first described fossil species of the genus and also of the subfamily Ptilodactylinae. Our discovery sheds further light on the paleodiversity and evolution of the family as well as on the faunal composition of the European Eocene amber forests.

**Abstract:**

The beetle family Ptilodactylidae contains more than 500 extant species; however, its fossil record is scarce and remains understudied. In this study, we describe a new species of Ptilodactylidae, *Ptilodactyla eocenica* Kundrata, Bukejs and Blank, sp. nov., based on a relatively well-preserved specimen from Baltic amber. We use X-ray microcomputed tomography to reconstruct its morphology since some of the principal diagnostic characters have been obscured by opaque bubbles. It is the third ptilodactylid species described from Baltic amber, and the first one belonging to the subfamily Ptilodactylinae. Additionally, we summarize the classification, diversity, and distribution of both extinct and extant Ptilodactylidae.

## 1. Introduction

Fossils play an important role in our understanding the origins, past diversity, and evolution of various organisms as well as the composition of paleoecosystems that prospered on our planet many million years ago [1,2,3,4]. Bioinclusions in amber, which is a fossilized sticky tree resin, usually represent well-preserved and complete three-dimensional fossil remains that are relatively easily comparable with extant representatives. Recent advances in nondestructive imaging techniques such as the X-ray microcomputed tomography [5,6,7] enable the visualization of morphological features, even in specimens that are in a bad state of preservation [8,9,10]. These techniques were recently successfully applied in a number of paleontological studies dealing with various animal taxa, including both vertebrates [11,12] and invertebrates [13,14,15]. Regarding Coleoptera (beetles), the micro-CT was used in some studies focused on the Burmese amber [10,16], but it is especially successful in reconstructing the morphology of specimens from Baltic amber (including genitalia and fine morphology of specimens with body length under 1 mm) [17,18,19,20].

Ptilodactylidae (toe-winged beetles) are dryopoid beetles that usually live in riparian, semiaquatic, and aquatic habitats [21,22], with larvae often having various adaptations for survival underwater [22,23,24,25]. The monophyly of the family was questioned in many studies [26,27,28,29], and its composition and subfamilial classification is far from fully understood [21,22,30,31,32,33,34]. As currently defined, Ptilodactylidae contain many genera previously assigned to Dascillidae and Scirtidae [32,35] and also a single described species of former Podabrocephalidae [34]. The Ptilodactylidae are in urgent need of a complete revision. Stribling [32] revised the group and proposed many changes and several new genera, but his Ph.D. work has remained unpublished in the sense of the ICZN [36]. Currently, more than 500 described and numerous undescribed species assigned to 29 genera and five subfamilies are included in Ptilodactylidae, with the vast majority of them known from the tropical and subtropical regions (Table A1). Some studies showed that some of the species originally described in Ptilodactylidae might in fact belong to other beetle families [32,37,38].

The fossil record of this family is rather scarce and was critically reviewed by Chatzimanolis et al. [39] and Alekseev and Jäch [40]. Motschulsky [41] described *Ptilodactyloides stipulicornis* Motschulsky, 1856 from Eocene Baltic amber; however, the short description is not informative enough to assign it with confidence to any subfamily nor even to Ptilodactylidae [40]. Chatzimanolis et al. [39] described *Aphebodactyla rhetine* Chatzimanolis et al., 2012 from the mid-Cretaceous Burmese amber. The subfamily placement of that species is uncertain because it shows a mosaic of characters of several ptilodactylid subfamilies. Additionally, Alekseev and Jäch [40] described *Electrolichas circumbalticus* Alekseev and Jäch, 2016 from Eocene Baltic amber, and classified it in the subfamily Anchytarsinae. Most recently, Kirejtshuk [42] described *Paralichas striatopunctatus* Kirejtshuk, 2019 (Cladotominae) from the Eocene Insect Limestone of the United Kingdom.

**Figure 1:**
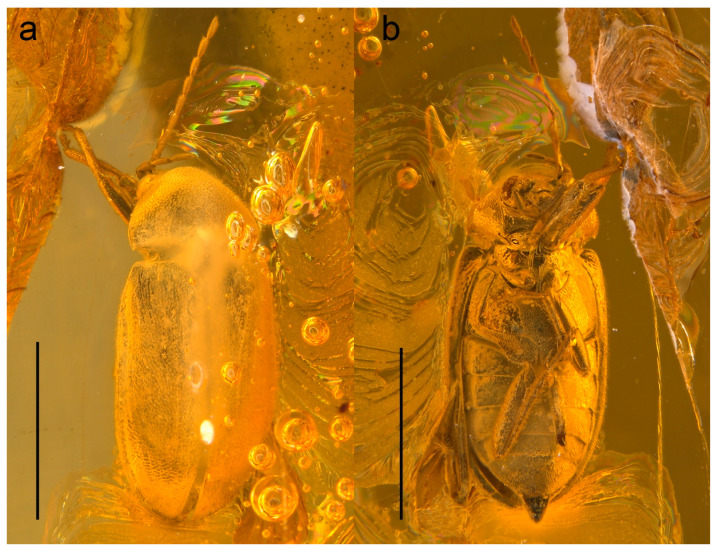
*Ptilodactyla eocenica* Kundrata, Bukejs and Blank, sp. nov., habitus. (**a**) Dorsal view; (**b**) ventral view. Scale bar = 3.0 mm.

In this study, we describe a new species of Ptilodactylidae based on a well-preserved specimen from Baltic amber. We used X-ray microcomputed tomography to reconstruct its morphology since some of the principal diagnostic characters have been obscured by opaque bubbles (Figure 1). Our results suggest that the studied specimen represents a new species of the otherwise extant, widespread genus *Ptilodactyla* Illiger, 1807. It is the first described fossil species of the genus and also of the subfamily Ptilodactylinae.

## 2. Materials and Methods

The amber piece was polished by hand, allowing improved views of the included specimen, and was not subjected to any additional treatment. For the purpose of light microscopic image capture, the amber specimen was fixed at a suitable angle of view to a Petri dish with gray plasticine modelling clay (Pelikan, Germany, No. 601492). It was photographed submersed in glycerol to prevent reflections and to reduce visibility of small scratches on the surface of the amber piece. Images were taken with a Leica MC 190 HD camera attached to a motorized Leica M205 C stereo microscope equipped with the flexible dome Leica LED5000 HDI or the conventional ring light Leica LED5000 RL-80/40 as an illuminator, applying the software Leica Application Suite X (version 3.7.2.22383, Leica Microsystems, Switzerland). Stacks of photographs were combined with the software Helicon Focus Pro (version 7.6.4, Kharkiv, Ukraine), applying the depth map or weighted average rendering methods.

The X-ray micro-CT (μCT) observations were conducted at Daugavpils University, Daugavpils, Latvia using a Zeiss Xradia 510 Versa system. Scans were performed with a polychromatic X-ray beam using energy set to 30 kV and power of 2 W. Sample detector distance was set to 32 mm and source to sample distance 44.39 mm. Tomographic slices were generated from 1601 rotation steps through 360-degree rotation, using 4X objective. Exposure time during each projection was set to 23 s. Variable exposure was set to four times at the thickest part of the amber to achieve similar amounts of photon throughput over whole sample. Acquired images were binned (2 × 2 × 2) giving a voxel size of 3.9 μm. Since the examined specimen was longer than the field of view for selected parameters, we carried out image acquisition using automated vertical stitch function for three consecutive scans with identical scanning parameters. The field of view between those scans was set to overlap 53% of data between adjacent fields of view. Images were imported into Dragonfly PRO (version 2020.2) software platform for interactive segmentation and 3D visualization. Prior to the full scan, 1-h warmup scan was conducted with identical stitch parameters but with reduced rotational steps (201) and exposure time set to 5 s. Volume rendering of X-ray microtomography of habitus is available as Supplement Video S1 following the recommendations of the best practice in the field [43].

The classification of Ptilodactylidae follows Bouchard et al. [44] and Chatzimanolis et al. [39], with subsequent changes by Kundrata et al. [34]. Numbers of species in ptilodactylid genera in Table A1 were compiled from Stribling [32] and various subsequent sources [21,24,34,37,38,45,46,47,48,49,50,51,52,53,54,55]. Morphological terminology follows Lawrence [22]. The holotype is deposited in the collection of the Department of Paleontology of the National Museum, Prague, Czech Republic (NMPC). The ZooBank LSID number for this publication is urn:lsid:zoobank.org:pub:B03EDD30-D551-41F1-A2DD-0D356A85873A (20 August 2021).

## 3. Results

### Systematic Paleontology

Order Coleoptera Linnaeus, 1758
Suborder Polyphaga Emery, 1886
Series Elateriformia Crowson, 1960
Superfamily Dryopoidea Billberg, 1820
Family Ptilodactylidae Laporte, 1838
Subfamily Ptilodactylinae Laporte, 1838
Genus *Ptilodactyla* Illiger, 1807

Type species. *Ptilodactyla elaterina* Illiger, 1807 (syn. of *Pyrochroa nitida* DeGeer, 1775). For more information, including synonyms, see Stribling [32] and Chatzimanolis et al. [39].

Diagnosis. Male antennae pectinate, with articulated rami; lateral pronotal carina anteriorly incomplete; trochantins concealed; pseudotetramerous tarsi with tarsomere IV reduced and tarsomere III lobed ventrally; claws with basal tooth; scutellar shield usually heart-shaped; apical palpomeres mostly sclerotized and securiform [32].

Composition. Approximately 370 described and many undescribed species [32].

Distribution. Worldwide; not native to present-day Europe [32] (Table A1). Exotic species found in the western Palearctic [56,57,58,59].

**Figure 2:**
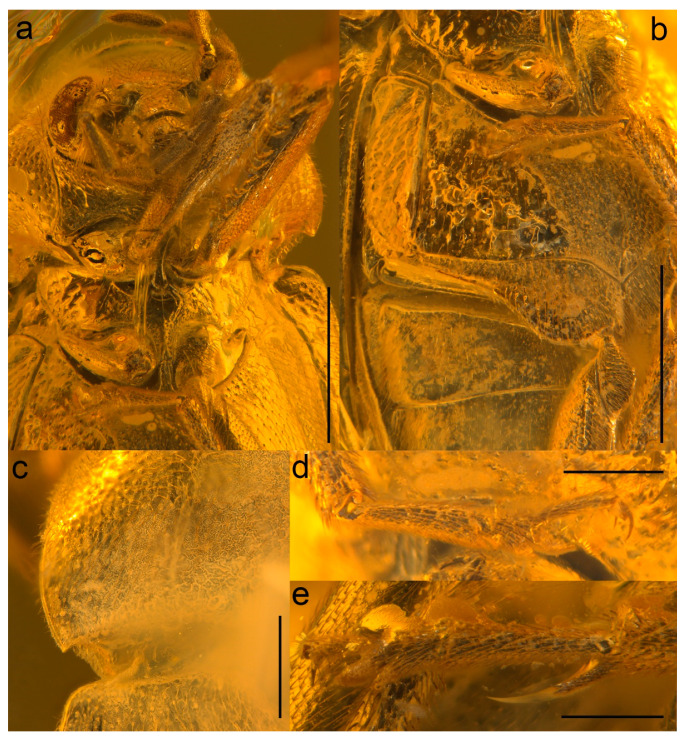
*Ptilodactyla eocenica* Kundrata, Bukejs and Blank, sp. nov., details of morphology. (**a**) Head and thorax; (**b**) metacoxal plate; (**c**) pronotum, dorsal view; (**d**) mesotarsus; (**e**) metatarsus. Scale bars = (**a**,**b**) 1.0 mm; (**c**) 0.5 mm; (**d**,**e**) 0.3 mm.

**Figure 3:**
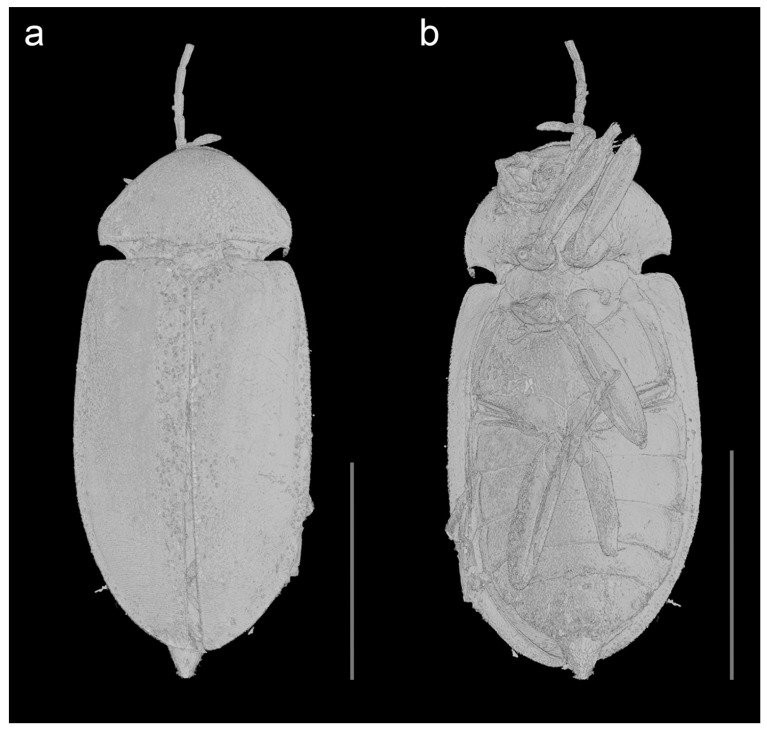
*Ptilodactyla eocenica* Kundrata, Bukejs and Blank, sp. nov., habitus. (**a**) Dorsal view; (**b**) ventral view. Scale bar = 4.0 mm.

**Figure 4:**
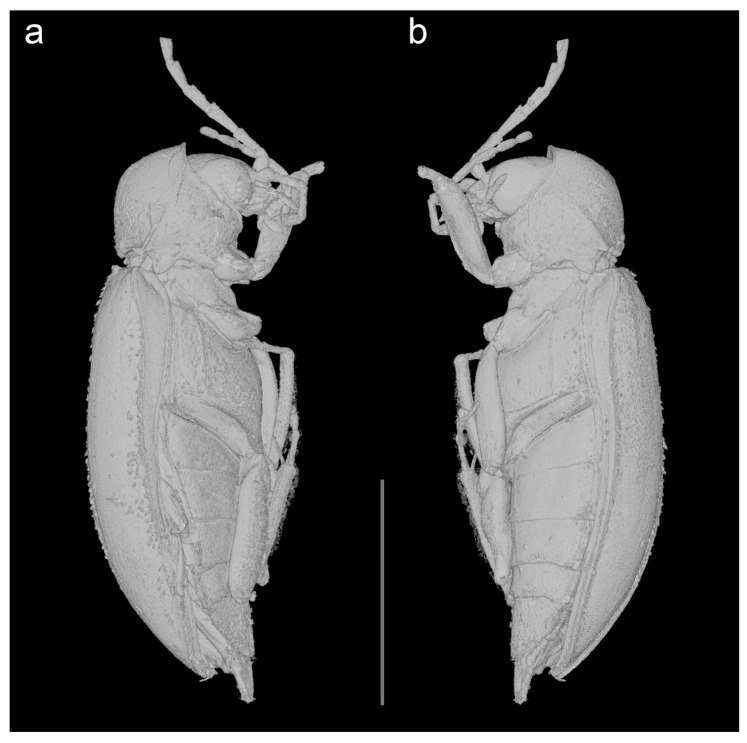
*Ptilodactyla eocenica* Kundrata, Bukejs and Blank, sp. nov., habitus. (**a,b**) Lateral views. Scale bar = 4.0 mm.

**Figure 5:**
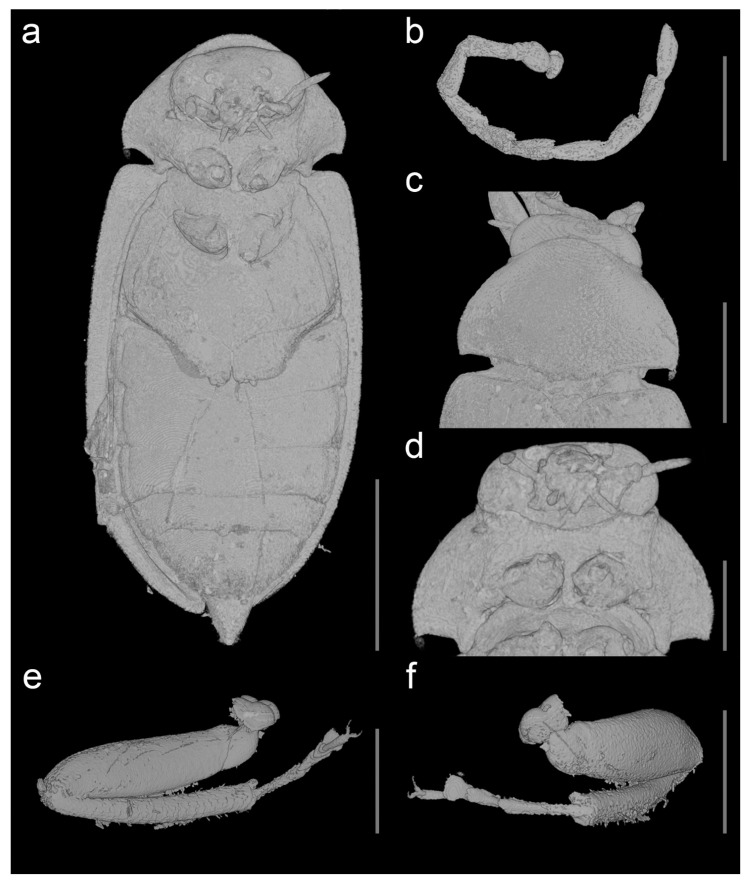
*Ptilodactyla eocenica* Kundrata, Bukejs and Blank, sp. nov., details of morphology. (**a**) Habitus, ventral view; (**b**) antenna, lateral view; (**c**) habitus, pronotum, and scutellar shield; (**d**) habitus, prosternum; (**e**,**f**) mesolegs. Scale bars = (**a**) 3.0 mm; (**b**,**d**–**f**) 1.0 mm; (**c**) 2.0 mm.

*Ptilodactyla eocenica* Kundrata, Bukejs and Blank, sp. nov.

urn:lsid:zoobank.org:act:DE2C5EE2-4CB6-40B3-86E6-A1848415589A; Figure 1, Figure 2, Figure 3, Figure 4 and Figure 5, Supplementary Video S1.

Type material. Holotype, female, NM-T 3472 (NMPC, ex coll. R. Kundrata, Olomouc, No. BAL0001, ex coll. J. Damzen, Lithuania, No. 8114). A complete beetle is included in a transparent, yellow amber piece with dimensions of 44 × 30 × 6 mm.

Type stratum and age. Mid-late Eocene, 48–34 Ma [60,61,62,63,64].

Type locality. Baltic Sea coast, Yantarny mine, Sambian (Samland) Peninsula, Kaliningrad Oblast, Russia.

Etymology. The specific epithet refers to the Eocene Epoch.

Diagnosis. This species is so far the only one described from the Baltic amber and can be recognized based on the following combination of characters: body about 6 mm long; antenna slightly serrate, reaching about third of elytra, antennomere 3 about 3.5 times as long as pedicel; terminal maxillary palpomere fusiform, about 4 times as long as wide, apically obliquely cut; pronotum about 1.6 times as wide as long; scutellar shield wider than long, heart-shaped, notched anteriorly; metacoxal plate well developed mesally and distinctly weaker laterally; abdominal ventrite 5 emarginate apically.

Description. Adult female. Body (Figure 1,Figure 3,Figure 4 and Figure 5a) about 6.1 mm long and 2.8 mm wide, strongly convex, about 2.2 times as long as wide; dorsally moderately densely setose.

Head (Figure 1b, Figure 2a, Figure 3b, and Figure 5a,d) declined, subquadrate, not visible in dorsal view, distinctly narrower than pronotum width. Eyes large, entire, strongly protuberant. Antennal insertions moderately widely separated. Frontoclypeal suture indistinct. Labrum strongly transverse, slightly convex, anteriorly slightly concave. Antenna (Figure 1, Figure 3, Figure 4, and Figure 5b) slightly serrate, reaching about third of elytra; scape robust, distinctly longer than pedicel; pedicel minute, slightly longer than wide; antennomere 3 about 3.5 times as long as pedicel, antennomeres 3–10 elongate, more than twice as long as wide, gradually widened toward apical part, with short serrations; terminal antennomere simple, elongate, apically narrowed, obliquely cut. Mandible basally broad, mesal, and apical parts not visible. Maxillary palpus moderately long; last three palpomeres elongate; terminal palpomere rather fusiform, about 4 times as long as wide, apically flattened and obliquely cut. Labial palpus distinctly shorter than maxillary palpus, with terminal palpomere fusiform.

Pronotum (Figure 1a, Figure 2c, Figure 3a, Figure 4, and Figure 5c) strongly convex, about 1.6 times as wide as than long (2.30 mm wide, 1.45 mm long), widest posteriorly; sides strongly curved; lateral carinae simple, anteriorly incomplete; anterior angles obtuse, not projecting, posterior angles short, weakly acute; posterior edge distinctly bisinuate and strongly crenulate, median part with more distinct median crenulation to fit anteriorly emarginate margin of scutellar shield; disc strongly convex, with rather rough surface covered with moderately dense rough punctures. Hypomeron (Figure 4) with distinct large punctures, punctures sparser and larger mesally. Prosternum (Figure 1b, Figure 2a, Figure 3b, and Figure 5a,d) strongly transverse, anteriorly widely concave, in front of coxa distinctly shorter than procoxal diameter; prosternal process short, reaching just behind procoxal cavities, parallel-sided but expanded apically, apex broadly rounded. Pronotosternal suture complete, simple, strongly curved. Scutellar shield (Figure 3a and Figure 5c) relatively small, wider than long, heart-shaped, notched anteriorly, rounded posteriorly. Elytra (Figure 1a, Figure 3a and 4) together about 1.6 times as long as wide (4.6 mm long, 2.8 mm wide) and 3.2 times as long as pronotum; convex, elongate-oval, widest just behind middle, conjointly rounded apically, with punctures fine and moderately dense, irregular, not forming distinct rows; elytral epipleuron complete, relatively wide, slightly narrowed behind metacoxae. Mesoventrite (Figure 1b, Figure 2a, Figure 3b, and Figure 5a,d) with anterior edge on same plane as metaventrite, procoxal rests shallow, mesoventral cavity shallow, mesoventral process well separated from metaventrite by suture. Mesocoxal cavities narrowly separated. Metaventrite wider than long, moderately convex; metanepisternum elongate, relatively wide, less than 3 times as long as wide, subparallel-sided. Metacoxae contiguous, extending laterally; metacoxal plate (Figure 1b, Figure 2b, Figure 3b, and Figure 5a) well-developed mesally and distinctly weaker laterally. Hind wing fully developed. Leg (Figure 1b, Figure 2a,d,e, Figure 3b, Figure 4, and Figure 5e,f) moderately long, femur robust, elongate; tibia slightly longer than femur, clothed with stiff, spinelike setae, particularly on outer edge, each tibia with pair of distinct spurs (less distinct on mesotibia). Tarsi pseudotetramerous. Tarsomere I almost 3 times as long as tarsomere II, tarsomeres II apically widened, tarsomere III with wide ventral lobe, tarsomere IV reduced, apical tarsomere simple, slender, elongate; claws basally toothed, moderately curved.

Abdomen (Figure 1b, Figure 3b, Figure 4 and Figure 5a) with five ventrites, with surface rather smooth, moderately densely covered by fine punctures; ventrites 1–4 with short lateral projections; ventrite 5 about twice as long as ventrite 4, widely subtriangular, slightly emarginate apically. Pygidium visible, subtriangular, gradually narrowed toward apex, narrowly rounded apically.

## 4. Discussion

The fossil record of Ptilodactylidae dates back to the Mesozoic; undescribed material is known from the Jurassic of China [65], the Lower Cretaceous outcrops in Spain [66], and the Lower Cretaceous Lebanese amber [67,68], and one described species and many undescribed are included in the mid-Cretaceous Burmese amber [39] (R. Kundrata, pers. observ.). Additionally, Kirejtshuk et al. [42] suggested that some Mesozoic specimens described in *Artematopodites* Ponomarenko, 1990 could represent Ptilodactylidae. The Eocene fossil record of the family includes the description of a single species from the Insect Limestone of the United Kingdom [42] and two ptilodactylid species from Baltic amber.

Baltic amber contains the most diverse assemblage of fossil insects to date [2,69,70]. Despite its popularity among paleontologists and taxonomists, and hence also a growing number of publications on the Baltic amber bioinclusions, there have been uncertainties regarding the origin and age of this amber [70,71,72,73]. More than 3000 animal species have been reported from Baltic amber to date [1,74], of which almost 500 belong to beetles [73]. Regarding Ptilodactylidae, only a single species described by Motschulsky [41] and representing a monotypic genus, i.e., *Ptilodactyloides stipulicornis*, was previously known from Baltic amber [69,75,76] until 2016, when Alekseev and Jäch [40] described additional ptilodactylid species in another monotypic genus, i.e., *Electrolichas circumbalticus*. Here described ptilodactylid species from Baltic amber shares the diagnostic characters with extant species of *Ptilodactyla*. The discovery of a Baltic amber fossil ptilodactylid that can be attributed to the extant genus is not surprising since approximately half of the fossil animals known from the relatively young Eocene European ambers represent extant genera [69].

The genus *Ptilodactyloides* was originally compared to *Ptilodactyla* and was considered similar to it but differing in the length and structure of antennae (surpassing apex of abdomen, and with a vertical appendage on each of antennomeres III–X) [41]. On the other hand, *Electrolichas* was placed by its authors in subfamily Anchytarsinae [40]. Alekseev and Jäch [40] considered the subfamilial and even familial placement of *Ptilodactyloides* uncertain. We failed to locate the type material, which might have been lost or even destroyed [40], and therefore we could not directly compare it with the here described species of *Ptilodactyla*. In Ptilodactylidae, males often have distinctly pectinate antennae while females have serrate antennae, which would suggest that the holotype of *Ptilodactyloides stipulicornis* is a male, and that of *Ptilodactyla eocenica* Kundrata, Bukejs and Blank, sp. nov. is a female (Figure 1, Figure 3, Figure 4, and Figure 5b). Even if we accept that *Ptilodactyloides* might be in fact a male of some *Ptilodactyla* (although it is not clear at all from the Motschulsky’s drawing), it differs considerably from *P. eocenica* Kundrata, Bukejs and Blank, sp. nov. in the body size (“Long. 1 lign.” in the original description [41], while 1 lign. (line) was usually around 2.5 mm in the year of description although it varied between countries [40]). Anyway, the true identity of *Ptilodactyloides stipulicornis* remains unclear. Although there might be a difference in body size between males and female in extant species of *Ptilodactyla*, it is usually only minimal (R. Kundrata, pers. observ., M. Ivie, pers. comm.). The discovery of a male of the here described *Ptilodactyla* species would help us to understand the extent of sexual dimorphism in that species as well as to better evaluate the differences between *Ptilodactyla eocenica* Kundrata, Bukejs and Blank, sp. nov. and *Ptilodactyloides stipulicornis*.

## Data Availability

The holotype of *Ptilodactyla eocenica* sp. nov. is deposited in the collection of the Department of Paleontology of the NMPC. Volume rendering of X-ray microtomography of habitus is available as Supplement Video S1.

## Supplementary Materials

The following is available online at https://www.mdpi.com/article/10.3390/biology10090877/s1, Video S1: *Ptilodactyla eocenica*, holotype, X-ray micro-CT volume rendering of the habitus.

## Appendix A

**Table A1:** Classification, diversity, and distribution of Ptilodactylidae. Most genera are in urgent need of revision so the generic classification and numbers of described species may change dramatically in future, also considering the undescribed genera reported by Stribling [32]. * = extinct taxon.

Subfamily	Genus	Distribution	Number of Species
Anchytarsinae Champion, 1897	*Anchycteis* Horn, 1880	Japan, USA	4 spp.
	*Anchytarsus* Guérin-Méneville, 1843 = *Tetraglossa* Champion, 1897	Americas	5 spp.
	*Byrrocryptus* Broun, 1893	Australia, New Zealand	5 spp.
	*Daemon* Laporte, 1836 = *Colobodera* Klug, 1838	Afrotropical realm	32 spp.
	*Electrolichas* Alekseev and Jäch, 2016	Europe: Baltic amber	1 sp.*
	*Epilichas* White, 1859	East and Southeast Asia	10 spp.
	*Lycomimodes* Lawrence and Ślipiński, 2013	Australia	1 sp.
	*Pseudoepilichas* Armstrong and Nakane, 1956	Japan	2 spp.
Aploglossinae Champion, 1897	*Aploglossa* Guérin-Méneville, 1849	South and Central America	11 spp.
	*Bradytoma* Guérin-Méneville, 1843 = *Brithycera* Erichson, 1847	South America	3 spp.
Araeopidiinae Lawrence, 1991	*Araeopidius* Cockerell, 1906	North America	1 sp.
Cladotominae Pic, 1914	*Austrolichas* Lawrence and Stribling, 1982	Australia	1 sp.
	*Cladotoma* Westwood, 1837 = *Telon* Champion, 1897	South America	7 spp.
	*Drupeus* Lewis, 1895	East Asia	6 spp.
	*Hovactyla* Fairmaire, 1901	Madagascar	1 sp.
	*Paralichas* White, 1859 = *Eucteis* Guérin-Méneville, 1861 = *Odontonyx* Guérin-Méneville, 1843	East Asia, North America, Madagascar; Eocene of the UK	11 spp. (1 *)
	*Pseudocladotoma* Pic, 1918	East and Southeast Asia	4 spp.
Ptilodactylinae Laporte, 1838	*Chelonariomorphus* Pic, 1916	Bolivia	1 sp.
	*Lachnodactyla* Champion, 1897	Central and North America	4 spp.
	*Lomechon* Wasmann, 1897	Costa Rica	1 sp.
	*Microdrupeus* Nakane, 1993	Japan	1 sp.
	*Pherocladus* Fairmaire, 1881	Southeast Asia, Fiji	9 spp.
	*Podabrocephalus* Pic, 1913	India	1 sp.
	*Ptilodactyla* Illiger, 1807 = *Daemonimus* Pic, 1953 = *Falsodaemon* Pic, 1913 = *Hypselothorax* Kirsch, 1866 = *Stenactyla* Fairmaire, 1896 = *Theriomorphus* Pic, 1913	worldwide, not native to West Palearctic; Baltic amber	ca. 370 spp. (1 *)
	*Stirophora* Champion, 1897 = *Chaetodactyla* Champion, 1897	Central and South America	2 spp.
*Incertae sedis*	*Aphebodactyla* Chatzimanolis, Cashion, Engel and Falin, 2012	Myanmar: Burmese amber	1 sp.*
	*Falsoptilodactyla* Pic, 1958	Afrotropical realm	1 sp.
	*Falsotherius* Pic, 1913	Southeast Asia	3 spp.
	*Octoglossa* Guérin-Méneville, 1843 = *Astyloglossa* Pic, 1919	Central and South America	4 spp.
	*Ptilodactyloides* Motschulsky, 1856	Europe: Baltic amber	1 sp.*
	*Therius* Guérin-Méneville, 1849	Afrotropical realm	7 spp.
	*Valoka* Deléve, 1872	Papua New Guinea: Bismarck Archipelago	1 sp.
